# The BARRIERS scale -- the barriers to research utilization scale: A systematic review

**DOI:** 10.1186/1748-5908-5-32

**Published:** 2010-04-26

**Authors:** Kerstin Nilsson Kajermo, Anne-Marie Boström, David S Thompson, Alison M Hutchinson, Carole A Estabrooks, Lars Wallin

**Affiliations:** 1 Clinical Research Utilization (CRU), Karolinska University Hospital, Eugeniahemmet T4:02, SE-171 76 Stockholm, Sweden; 2 Knowledge Utilization Studies Program (KUSP), Faculty of Nursing, University of Alberta, 5-104 Clinical Science Building, Edmonton, Alberta T6G 2G3, Canada; 3 Department of Neurobiology, Care Sciences and Society, Karolinska Institutet, Alfred Nobels Allé 23, 23 300, SE-141 83 Huddinge, Sweden; 4 Northern Ontario School of Medicine, 955 Oliver Road, Thunder Bay, Ontario P7B 5E1, Canada; 5 School of Nursing, Deakin University and Cabrini-Deakin Centre for Nursing Research, Cabrini Institute, 183 Wattletree Road Malvern 3144, Victoria, Australia

## Abstract

**Background:**

A commonly recommended strategy for increasing research use in clinical practice is to identify barriers to change and then tailor interventions to overcome the identified barriers. In nursing, the BARRIERS scale has been used extensively to identify barriers to research utilization.

**Aim and objectives:**

The aim of this systematic review was to examine the state of knowledge resulting from use of the BARRIERS scale and to make recommendations about future use of the scale. The following objectives were addressed: To examine how the scale has been modified, to examine its psychometric properties, to determine the main barriers (and whether they varied over time and geographic locations), and to identify associations between nurses' reported barriers and reported research use.

**Methods:**

Medline (1991 to September 2009) and CINHAL (1991 to September 2009) were searched for published research, and ProQuest^® ^digital dissertations were searched for unpublished dissertations using the BARRIERS scale. Inclusion criteria were: studies using the BARRIERS scale in its entirety and where the sample was nurses. Two authors independently assessed the study quality and extracted the data. Descriptive and inferential statistics were used.

**Results:**

Sixty-three studies were included, with most using a cross-sectional design. Not one study used the scale for tailoring interventions to overcome identified barriers. The main barriers reported were related to the setting, and the presentation of research findings. Overall, identified barriers were consistent over time and across geographic locations, despite varying sample size, response rate, study setting, and assessment of study quality. Few studies reported associations between reported research use and perceptions of barriers to research utilization.

**Conclusions:**

The BARRIERS scale is a nonspecific tool for identifying general barriers to research utilization. The scale is reliable as reflected in assessments of internal consistency. The validity of the scale, however, is doubtful. There is no evidence that it is a useful tool for planning implementation interventions. We recommend that no further descriptive studies using the BARRIERS scale be undertaken. Barriers need to be measured specific to the particular context of implementation and the intended evidence to be implemented.

## Background

The call to provide evidence-based nursing care is based on the assumption that integrating research findings into clinical practice will increase the quality of healthcare and improve patient outcomes. Reports of the degree to which nurses base their practice on research have been discouraging [1-12]. Despite efforts to increase research use, translating research findings into clinical practice and ensuring they are implemented and sustained remains a challenge. A strategy commonly recommended for bridging the gap between research and practice is to identify barriers to practice change [13,14] and then implement strategies that account for identified barriers. Typically, barriers are context-dependent; therefore, implementation strategies should be tailored according to the context and the specific barriers identified [15]. Some evidence supports this approach, although little is known about which barriers are valid, how these barriers should be identified, or what interventions are effective for overcoming specific barriers.

In nursing, the BARRIERS scale, developed by Funk *et al. *and published in 1991 [16], has been used extensively to identify barriers to research use. Investigators have used this instrument since then, compiling a corpus of research findings that documents barriers to research use across continents, time, and study settings. This sustained research effort presents a unique opportunity to examine trends in the results.

### The BARRIERS scale

Funk *et al. *developed the BARRIERS scale to assess clinicians', administrators', and academicians' perceptions of barriers to the use of research findings in practice [16]. Respondents are asked to rate the extent to which they perceive each statement (item) as a barrier to the use of research findings. Items are rated on a four-point scale (1 = to no extent, 2 = to a little extent, 3 = to a moderate extent, 4 = to a great extent); respondents can also choose a no opinion alternative. In addition to rating the barrier items, respondents are invited to add and score other possible barriers, to rank the three greatest barriers, and to list factors they perceive as facilitators of research utilization. The scale items were developed from literature on research utilization, the Conduct and Utilization of Research in Nursing (CURN) project questionnaire [17], and data gathered from nurses. Potential items were assessed by a group of experts. Items demonstrating face and content validity were retained and then pilot-tested. This led to minor rewording of some items and the inclusion of two additional items, resulting in a scale consisting of 29 items representing potential barriers to research utilization [16].

In the psychometric study by Funk *et al.*, 1,989 nurses representing five educational strata responded to the scale (response rate 40%) [16]. Exploratory factor analysis (principal component analysis with varimax rotation) was performed to investigate underlying dimensionality of the scale. The sample was divided in two subsamples, and the analyses were performed on the two halves. The two subsamples produced similar four-factor solutions with 28 items with loadings of 0.40 or greater on one factor. One item (namely, the amount of research is overwhelming) did not load distinctly on any of the factors and was subsequently removed from the scale. Finally, a factor analysis was performed on the entire sample, resulting in the same four-factor solution. Thus, the final scale consisted of 28 items. Funk *et al. *reported a four-factor solution and considered these four factors, or subscales, to be congruent with the factors in Rogers' diffusion of innovation theory [18]. The subscales were labeled: the characteristics of the adopter, such as the nurse's research values, skills, and awareness (eight items); the characteristics of the organization, such as setting barriers and limitations (eight items); the characteristics of the innovation, such as qualities of the research (six items); and the characteristics of the communication, such as presentation and accessibility of the research (six items) (Table 1). Consistent with Funk *et al. *[16,19,20], we refer to the individual subscales as the nurse, setting, research, and presentation subscales. In Funk's psychometric article, Cronbach's alpha values for the four subscales were 0.80, 0.80, 0.72, and 0.65, respectively [16]. To test the temporal stability of the instrument, 17 subjects answered the questionnaire twice, one week apart. Pearson product moment correlations between the two data sets ranged from 0.68 to 0.83, which according to the authors indicated acceptable stability [16].

**Table 1:** Rank order of barriers (n = 53 studies). The items ranked among the top ten in most studies are italicized.

Subscale and Item	Range in percentage of nurses rating the item as a moderate to great barrier	Number of studies with > 50% of nurses rating the item as a moderate to great barrier	Number of studies rating the item among the top ten of barriers
**Nurse Subscale: The nurse's research values, skills and awareness**			

*The nurse is unaware of the research*	10-77	24	27
*The nurse does not feel capable of evaluating the quality of the research*	5-83	25	25
The nurse is isolated from knowledgeable colleagues with whom to discuss the research	16-89	20	16
The nurse is unwilling to change/try new ideas	3-59	6	2
The nurse sees little benefit for self	3-61	5	2
There is not a documented need to change practice	8-55	1	2
The nurse feels the benefits of changing practice will be minimal	5-57	6	1
The nurse does not see the value of research for practice	3-58	3	0

**Setting Subscale: Setting barriers and limitations**			

*There is insufficient time on the job to implement new ideas*	16-89	38	49
*The nurse does not have time to read research*	8-88	38	48
*The nurse does not feel she/he has enough authority to change patient care procedures*	22-85	33	43
*The facilities are inadequate for implementation*	16-88	32	36
*Other staff are not supportive of implementation*	13-79	29	31
*Physicians will not cooperate with implementation*	11-83	26	31
The nurse feels results are not generalizable to own setting	6-79	23	24
Administration will not allow implementation	9-71	8	7

**Research Subscale: Qualities of the research**			

The research has not been replicated	4-67	12	6
The literature reports conflicting results	1-72	7	5
The research has methodological inadequacies	5-67	4	5
Research reports/articles are not published fast enough	9-69	5	4
The nurse is uncertain whether to believe the results of the research	3-55	4	0
The conclusions drawn from the research are not justified	0-57	1	0

**Presentation Subscale: Presentation and accessibility of the research**			

*The statistical analyses are not understandable*	4-90	36	40
*The relevant literature is not compiled in one place*	8-86	33	37
Research reports/articles are not readily available	23-94	19	18
Implications for practice are not made clear	10-82	19	17
The research is not reported clearly and readably	3-83	18	15
The research is not relevant to the nurse's practice	5-60	3	0
**Items not included in any of the subscales**			
The amount of research information is overwhelming* (27 articles)	10-71	11	13
Research reports/articles are written in English** (15 articles)	18-89	6	11

*Did not load on any of the four factors (subscales) in Funk *et al.*'s factor analysis

**Additional item in 15 studies from non-English-speaking countries

Two previous reviews of the BARRIERS scale have been published [21,22]. These reviews were primarily descriptive; their results suggest relative consistency in the ratings of barriers across included studies. The systematic review reported here differs from these two reviews in three ways: we assess the quality of included studies; we analyze the BARRIERS scale literature and discuss the validity of the scale using both individual items and the four BARRIERS subscales; and we provide a comprehensive, in-depth analysis of trends, concordance between studies, and associations between the results and the study characteristics.

The aim of this systematic review was to examine the state of knowledge resulting from use of the BARRIERS scale and, secondarily, to make recommendations about future use of the scale. The specific research objectives addressed were as follows:

1. To examine how the scale has been modified.

2. To examine psychometric properties of the scale.

3. To determine the main barriers, over time, and by geographic location.

4. To identify associations between nurses' reported barriers and reported research use.

## Methods

### Search strategy

**Figure 1:**
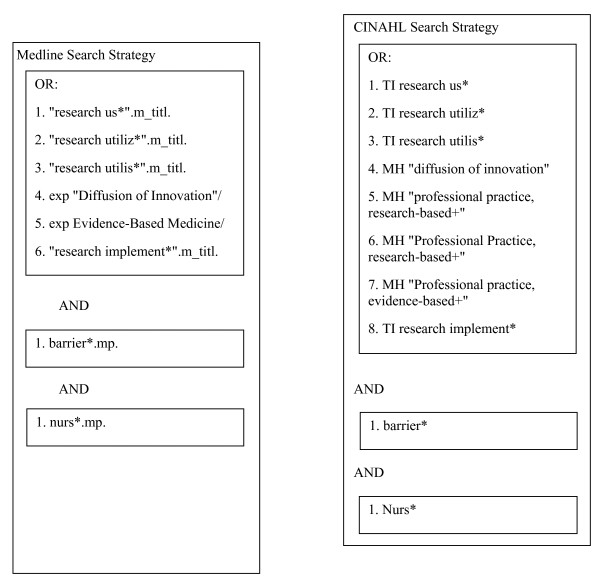
**Search strategy**.

We searched for published reports in Medline (1991 to 2007) and the Cumulative Index to Nursing and Allied Health Literature (CINAHL) (1991 to 2007) using the search terms outlined in Figure 1. We searched for unpublished dissertations in ProQuest^® ^Digital Dissertations (1991 to 2007) using a title search of 'research' and 'barriers'. Additionally, we conducted a citation search for Funk *et al.*'s original 1991 BARRIERS scale article [16] using Scopus. Finally, we conducted ancestry searches on relevant studies and two published reviews [21,22]. Grey literature was not included in the search strategy. In October 2009, using the same databases and search terms, the search was updated for the period from 1 January 2008 to 30 September 2009.

### Inclusion criteria

A study was eligible for inclusion if the study used Funk *et al.*'s BARRIERS scale in its entirety and the study sample was nurses. For criterion one, we included studies that used the original BARRIERS scale or applied minor modifications to the original scale (*i.e.*, word modification). For criterion two, we included all types of registered nurses or student nurses regardless of role (*i.e.*, administrator, educator, staff nurse) or setting (*i.e.*, acute care, community care, long-term care). Only studies in English or a Scandinavian language (*i.e.*, Swedish, Danish, or Norwegian) were included, reflecting our team's language abilities. No restrictions were made on the basis of study design.

### Screening process

**Figure 2:**
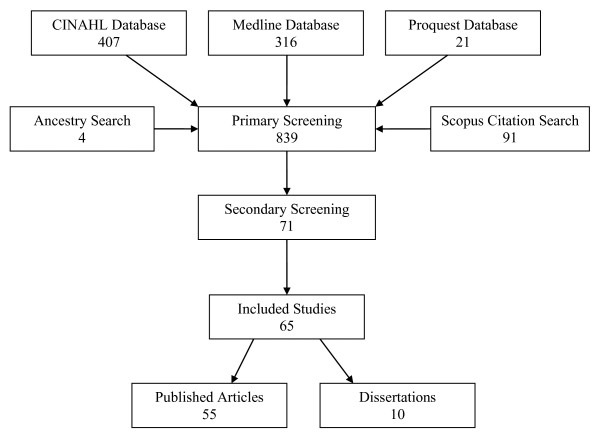
**Search and retrieval process**. -Figure includes BOTH Barta Thesis and Barta manuscript. -Figure includes BOTH Baernholdt thesis and Baernholdt manuscript -Ancestry search includes: Green Thesis, Doerflinger Thesis, Nilsson Kajermo Thesis, Niederhauser & Kohr paper (these are the included citations that were not found by the search)

The original search resulted in 605 citations. One member of the team used the inclusion criteria to assess the titles, abstracts, and reference lists of the articles. This resulted in 60 citations. Secondary screening excluded six studies because only select items from the BARRIERS scale were used. Overall, screening resulted in 44 published articles and 10 dissertations, representing 52 studies (Figure 2). The updated search returned 234 additional citations and screening resulted in 11 new articles (Figure 2). For three authors (Barta, Baernholdt, and Nilsson Kajermo), both their dissertations [23-25] and articles published [26-30] from the dissertations were included because the dissertations presented results that were not reported in the articles. We could not locate any published papers from seven dissertations.

### Quality assessment

The included studies (Table 2) were assessed for methodological strength using two quality assessment tools: one for cross-sectional studies, and one for before-and-after intervention design. These tools have been used in a previous review [31], but we modified the tools slightly because the same instrument (*i.e.*, BARRIERS scale) was used in all the studies. We omitted two questions pertaining to measurement of the dependent variable. The modified quality assessment tool for cross-sectional studies included 11 questions (Table 3). The tool for before-and-after studies included 13 questions (Table 4). Each question was scored with 1 if the stated criterion for the question was met and with 0 if the stated criterion was not met. There was also a not applicable alternative. The actual score was calculated and divided by the total possible score. The maximum score for both the cross-sectional and the before-and-after studies tools was 1. A score <0.50 was considered weak quality, 0.50 to 0.74 moderate quality, and ≥0.75 strong quality.

**Table 2:** Characteristics of included studies in chronological order

Authors and year	Country	Setting/speciality	Sample	Quality	Sample size/(response rate %)	No opinion reported
Funk *et al. *1991	USA	Mixed	Clinical nurses	moderate	924/(40)	No

Barta 1992, 1995	USA	Mixed/Paediatric care	Educators	moderate	213/(52)	No

Shaffer 1994	USA	Hospitals/Critical care	RN	moderate	336/(42)	No

Funk *et al. *1995	USA	Mixed	Clinical administrators	moderate	440/(40)	No

Bobo 1997	USA	Hospital	RN	weak	40/(-)	No

Carroll *et al. *1997	USA	Hospital and faculty	RN, advanced practice nurses, educators	weak	356/(30)	Yes

Dunn *et al. *1997	UK	Palliative, elderly care	CNS, nurses	moderate	316/(-)	Yes

Grap *et al. *1997	USA	Hospitals/Critical care	Staff nurses, managers, educators	moderate	353/(35.3)	No

Greene 1997	USA	Office practices	Oncology nurses	moderate	359/(36)	Yes

Lynn and Moore 1997	USA	Hospitals	Nurse managers	weak	40/(51)	No

Walsh 1997	UK	Hospitals/Emergency and Acute care	RN	weak	124/(62)	No

Walsh 1997	UK	Hospitals, community	RN	weak	141/(76.2)	No

Walsh 1997	UK	Community	RN	weak	58/(71)	No

Lewis *et al. *1998	USA	Mixed/Nephrology	Nurses	weak	498/(34)	No

Nilsson Kajermo *et al. *1998	Sweden	Hospitals	RN	moderate	237/(70)	Yes

^Nolan *et al. *1998	UK	Hospitals	Nursing staff	weak	382/(27)	No

Rutledge *et al. *1998	USA	Mixed/Oncology	Staff nurses, managers, CNS	strong	1100/(38) 407/(38)	Yes

Retsas and Nolan 1999	Australia	Hospitals	RN	weak	149/(25)	No

*Closs *et al. *2000	UK	Hospitals	Nurses	moderate	712/(36) 530/(35.4) 182/(37.3)	No

Nilsson Kajermo *et al. *2000	Sweden	Hospitals and faculty	Educators, students, administrators	moderate	36/(82) 166/(81) 33/(81)	Yes

†Parahoo 2000	Northern Ireland	Hospitals (general, psych and disability)	Staff nurses, specialist nurses, managers	moderate	1368/(52.6)	Yes

Retsas 2000	Australia	Hospital	RN	weak	400/(50)	No

*Closs and Bryar 2001 Factor analysis	UK	Hospitals, community, health authority	Nurses	moderate	2009/(44.6)	Yes

*Griffiths *et al. *2001	UK	Community	Nurses	moderate	1297/(51.5)	No

Johnson and Maikler 2001	USA	Hospitals/Neonatal intensive care unit	Neonatal nurses	moderate	132/(17.6)	No

^Marsh *et al. *2001	UK	Hospitals (1+2)	Qualified nursing staff	moderate	382/(27) 549/(36.4)	No

†Parahoo and McCaughan 2001	UK	Hospitals/Medical and surgical care	Nurses	weak	Med 210/(-) Surg 269/(-)	No

Oranta *et al. *2002	Finland	Hospitals	RN	moderate	253/(80)	Yes

*Bryar *et al. *2003	UK	Hospitals, community, health authority	Nurses	moderate	2009/(44.6)	No

Kuuppelomäki and Toumi 2003	Finland	Hospitals, community	RN	moderate	400/(67)	Yes

McCleary and Brown 2003	Canada	Hospital/Paediatric	Paediatric nurses	moderate	176/(33.3)	Yes

Mountcastle 2003	USA	Mixed	CNS	moderate	162/(40.5)	Yes

Sommer 2003	USA	University hospital	RN	moderate	255/(27.8)	Yes

Carolan Doerflinger 2004	USA	Acute care	Acute care nurse administrators	weak	86/(9)	Yes

Carrion *et al. *2004	UK	Mental Health	RN	moderate	47/(53.4)	Yes

Glacken and Chaney 2004	Ireland	Teaching and non- teaching hospitals	RN	weak	169/(39.6)	No

Hommelstad and Ruland 2004	Norway	Hospital/Perioperative	OR Nurses	moderate	81/(51)	Yes

Hutchinson and Johnston 2004	Australia	Teaching hospital	RN	moderate	317/(45)	Yes

Kirshbaum *et al. *2004	UK	Mainly hospitals/Breast cancer	Breast cancer nurses	moderate	263/(76.2)	Yes

LaPierre *et al. *2004	USA	Hospital/Perianesthesia	Staff nurses	weak	20/(67)	Yes

Nilsson Kajermo 2004	Sweden	Mixed	RN/Midwives educators administrators	moderate	1634/(51-82)	Yes

Patiraki *et al. *2004	Greece	General and oncology hospitals	Nurses	moderate	301/(72)	Yes

Ashley 2005	USA	Hospitals/Critical care	Critical care nurses	moderate	511/(17)	No

Baernholdt 2005, 2007	Various	Governments	Chief nursing officers	weak	38/(45)	No

Brenner 2005	Ireland	Not reported	Paediatric nurses	moderate	70/(35)	No

Fink *et al. *2005	USA	University hospital Magnet hospital	RN	weak	Pre 215/(24) Post 239/(27)	No

Niederhauser and Kohr 2005	USA	Paediatric	Paediatric nurse practitioners	strong	431/(69)	Yes

Paramonczyk 2005	Canada	Hospitals	RN (degree)	weak	25/(-)	No

Karkos and Peters 2006	USA	Community hospital (magnet hospital)	Licensed nursing staff	moderate	275/(47)	Yes

§Thompson *et al. *2006	China, Hong Kong	Mixed settings	RN	moderate	1487/(30)	No

Andersson *et al. *2007	Sweden	University hospitals/Paediatric care	RN, Paediatric nurses	moderate	56/(92)	Yes

Andersson *et al. *2007	Sweden	University hospitals/Paediatric care	RN, Trainee programme, specialist education in paediatric nursing Control	moderate	113/(80)	Yes

Atkinson and Turkel 2008	USA	Hospital (magnet hospital)	RN	weak	249/(23)	No

Boström *et al. *2008	Sweden	Elder Care	RN	moderate	140/(67)	Yes

§Chau *et al. *2008	China, Hong Kong	Mixed settings	RN	moderate	1487/(30)	yes

Deichmann Nielsen 2008	Denmark	Hospital	RN	weak	18/(81)	no

Mehrdad *et al. *2008	Iran	Teaching hospitals and Faculty	RN Educators	strong	375/(-) 35/(70)	yes

Nilsson Kajermo *et al. *2008	Sweden	University hospital	RN Midwives	moderate	833/(51)	no

Oh 2008	Korea	Teaching hospitals/Intensive and critical care	RN Nurse managers	weak	63/(-)	no

Brown *et al. *2009	USA	Academic medical centre	Nurses	moderate	458/(44.68)	Yes

Schoonover 2009	USA	Community hospital	RN	weak	79/(21)	yes

Strickland and O'Leary-Kelly 2009	USA	Mixed/Acute care	Educators	weak	122/(41)	yes

Yava *et al. *2009	Turkey	Teaching and Military Hospitals	Nurses	moderate	631/(66.6)	yes

Footnote: From four samples/studies (*, ^, †, §) ten articles were published

**Table 3:** Summary of quality assessment of included studies with cross-sectional design (n = 61)

	Number of studies		
Sampling:	**Yes**	**No**	N/A*
1. Was probability sampling used?	16	44	1
2. Are the participants likely to be representative of the target population?			
a) Very likely	2		
b) Somewhat likely	48		
c) Not likely	11		
3. Was sample size justified to obtain appropriate power?	53	8	
4. Was sample drawn from more than one site?	45	16	
5. If there are groups in the study, is there a statement they are matched in design or statistically adjusted?	10	28	23
6. Response rate more than 60%	16	45	

Measurement:			
1. Reliability indices	42	12	7
2. Factor analysis	14	19	28

Statistical analysis:			
1. Were p-values reported?	43	3	15
2. Were confidence intervals reported?	2	41	18
3. Were missing data managed appropriately?	27	34	

*N/A = not applicable

**Table 4:** Summary of quality assessment of included studies with before-and-after design (n = 2)

	Number of studies		
**Sampling**	**Yes**	**No**	**N/A**
1. Was probability sampling used?	1	1	
2. Was sample size justified to obtain appropriate power?	1	1	
3. Are the participants in the study likely to be representative of the target population?			
a. Very likely			
b. Somewhat likely	2		
c. Not likely			

**Design**			
1. One pretest or baseline and several posttest measures		2	
2. Simple before-and-after study			

**Control of confounders**:			
1. Does the comparison strategy attempt to create or assess equivalence of the groups at baseline?			
a. Yes, by matching		2	
b. Yes, by statistical adjustment		2	
c. No	2		
2. The group comparisons were the same for all occasions: (pre, baseline, and post evaluation)	1	1	

**Data collection and outcome measurement**			
1. Reliability indices	1	1	

**Statistical analysis**			
1. Was (were) the statistical test(s) used appropriate for the aim of the study?	2		
2. Were p-values reported?	2		
3. Were confidence intervals reported?		2	
4. Were missing data managed appropriately?		2	

**Drop outs** Is attrition rate < 30%?	1	1	

### Data extraction

A protocol was developed to obtain information about design, setting, sampling techniques, sample and sample size, response rate, additional questionnaires used, results of subscales and items rating, and factors linked to barriers. To validate the protocol, four of the authors read and assessed five papers independently. Agreement was achieved on how to use the protocol and to extract data. For data extraction, two authors read all the articles. Any discrepancies between the two authors were resolved by consensus.

### Data analysis

Descriptive statistics were calculated, including frequencies for the barrier items, mean values of the subscales (for studies reporting the subscales originally identified by Funk *et al. *[16]), and Spearman's rank order correlations.

To identify the top ten barriers for the studies reporting the ranked items, we calculated the frequencies with which each item was reported among the top ten barriers, thus deriving a total score per item (max 53 points = being among top ten in 53 studies that reported results on item level). Because some articles reported the whole and others reported on fractions of the same sample, we chose to include studies reporting the whole sample in this calculation [32-34], thereby excluding four articles reporting results from subsamples [35-38].

To compare the reported rank order of items, we used Spearman's rank order correlations, including studies that reported rank orders of all items. Given the large number of correlation tests, a p-value <0.01 was considered as statistically significant. In this analysis we included only articles reporting on the whole study sample [32-34]. For articles reporting rank order and percentage of agreement with the barriers statement for more than one subsample, but not for the total sample [28,39,40], we calculated weighted mean percentage values for agreement with the barrier statements (by multiplying each subsample size by the reported subsample percentage, summing the scores, and then dividing by the total sample size). The weighted mean percentage values were then used to create a rank order for the total sample.

For the top ten items identified for the time periods (1991 to 1999 and 2000 to September 2009), we compared, using Student's t-test for independent samples, subscale means and mean percentages for agreement with the barrier statements. We also compared subscale means and mean percentages for the top ten items between geographic locations (studies in North America, Europe-English, Europe non-English, Australia/Asia) using ANOVA and Bonferroni post hoc tests. Because of repeated tests, a p-value of <0.01 was considered as statistically significant.

## Results

Characteristics of the 63 studies included in this review are presented in Table 2[19,20,23-28,30,32-39,41-70][12,29,40,71-85].

### Quality of included studies

The assessed quality of the included articles and dissertations ranged from 0.27 to 0.78, resulting in quality being judged as weak for 22 studies, moderate for 38 studies, and strong for three studies (Table 2). Less than one-half of the included studies used probability sampling or achieved a response rate exceeding 60% (Table 3 and 4). Thirty-six studies failed to report on missing data and/or no opinion responses (Table 2, 3 and 4).

### Design

Two studies used a pre- and post-intervention design [42,76], one study was a methodological study [47], and two studies used multivariate regression techniques [29,66]. In the remainder, cross-sectional, descriptive, and bivariate correlational designs were used.

### Sample

Sample sizes in the included studies ranged from 18 to 2009 (Table 2). In total, the current review is based on the results of 19,920 respondents. Ten studies reported a sample of more than 500 respondents; twelve studies reported a sample of less than 80 respondents. Response rates varied from 9% to 92%. The samples consisted of nurses with various role titles (*e.g.*, nurses, nurse clinicians, registered nurses, staff nurses), working in various specialties and settings (Table 2). In other studies, the samples consisted of nurse managers/administrators (n = 8), nurse educators/teachers (n = 6), clinical nurse specialists/advanced practice nurses (n = 4), government chief nursing officers (n = 1), and nursing students (n = 1) (Table 2). Seventy-one percent of the studies (n = 45) were conducted in the United States, Canada, United Kingdom, Ireland, or Australia (Table 2). One study comprised an international sample of chief nursing officers, representing various countries and mother tongues [23,26].

### Modifications of the scale

Both the original 29-item BARRIERS scale and the 28-item version were represented in the included studies.

#### Modification of language

In eight studies, minor changes in the wording of the statements were made, mainly according to British language style [32,33,36,45,49,68-70]. Lynn and Moore [59], Kuuppelomäki and Tuomi [56], and Baernholdt [23,26] chose to use the word 'I' instead of 'nurse' in the statements. For example, the item 'the nurse is unaware of the research' was reworded to read 'I am unaware of the research.' The BARRIERS scale was translated to Swedish [12,25,28-30,40,71], Finnish [56,62], Greek [63], Norwegian [52], Danish [75], Persian [78], Turkish [85], Korean [80], and Cantonese Chinese [74,84].

#### Modifications of item and response format

In two articles, the twenty-sixth item in the BARRIERS scale ('the nurse is unwilling to change/try new ideas') was divided into two items: 'the nurse is unwilling to change practice' and 'the nurse is unwilling to try new ideas' [74,84]. In two studies, the 'no opinion' response option was changed to 'do not know' or 'neither agree nor disagree' and was reordered in the answer options [56,59]. In two further studies, the 'no opinion' response option was reordered to the center of the scale [53,84].

#### Barriers related to specific research findings

Respondents were asked to indicate the extent to which they perceived barriers to use of specific research findings in the studies by Grap *et al. *(hemodynamic monitoring) [50], Greene (guideline for pain management) [51], Carolan Doerflinger (use of restraints) [44], and Baernholdt (the impact of nurse staffing on patient and nurse outcomes) [23,26].

#### The 'no opinion' response category

In 32 of the included studies, the authors reported the frequency or percentage of 'no opinion' responses (Table 2). In all these studies, the highest numbers or percentages of 'no opinion' responses were for items belonging to the research subscale. In some studies, more than one-half of the respondents chose the 'no opinion' alternative for some of the items in this subscale [12,25,28,30,40,52,56,71], which the authors interpreted as an indication of lack of knowledge of research methods.

### Reports on psychometric properties

#### Reliability

Fourteen studies reported Cronbach's alpha values for the total scale, with scores ranging from 0.84 to 0.96, indicating internal consistency [30,40,45,48,51,53,57,62,64,71,74,78,84,85]. The Cronbach's alpha values for the subscales identified by Funk *et al. *[16] are presented in 24 studies and varied from 0.47 to 0.94 (Table 5). Of these, 18 studies reported alpha values below 0.70, mostly on the presentation subscale [12,19,20,25-28,39,45,46,48,51,52,57,63,73,76,84].

**Table 5:** Reported mean and/or Cronbach's alpha values on the Barrier Scale subscales nurse, setting, research, and presentation (n = 35).

Authors	Sample	Nurse (8 items) m	Setting (8 items) m	Research (6 items) m	Presentation (6 items) m	Cronbach's alpha
Funk *et al. *1991	Nurses	2.56	3.00	2.29	2.72	0.65-0.80

Funk *et al. *1995	Adm	2.78	2.86	2.35	2.80	0.65-0.80

Barta 1995	Educators	**2.98**	2.91	2.23	2.67	0.55-0.79

Carroll *et al. *1997	Mixed	2.3	2.7	2.2	2.6	0.67-0.81

Lynn and Moore 1997	NM	2.41	2.56	2.75	**3.11**	Not reported

Bobo 1997	PreIG	2.85	3.06	3.04	2.56	Not reported

	PreCG	2.91	**3.30**	**3.31**	2.83	

	PostIG	2.50	2.83	3.19	2.22	

	PostCG	2.84	3.23	3.14	2.88	

Dunn *et al. *1997	Nurses	Not reported	Not reported	Not reported	Not reported	0.4760-0.7796

Greene 1997	Nurses	**1.42**	**1.72**	**1.24**	**1.39**	0.69-0.83

Rutledge *et al. *1998	Nurses NM	1.82 2.60	2.52 2.69	2.04 2.23	2.53 2.58	0.69-0.79

Nilsson Kajermo *et al. *1998	RN	2.2	2.7	2.1	2.6	0.81-0.87

Parahoo 2000	Mixed	2.31	2.73	2.26	2.44	0.8368-0.8957

Nilsson Kajermo *et al. *2000	Educators	2.5	2.7	1.8	2.6	

	Stud	2.4	2.8	2.1	2.6	0.64-0.94

	Adm	2.6	2.5	2.1	2.7	

Oranta *et al. *2002	RN	2.35	2.72	2.28	2.62	0.7193-0.8080

Sommer 2003	RN	2.38	2.93	2.39	2.60	0.71-0.85

Mountcastle 2003	CNS	2.73	2.85	2.52	2.40	Not reported

McCleary and Brown 2003	Paediatric nurses	2.29	2.61	2.39	2.63	0.88-0.93

Carrion *et al. *2004	RNs	Not reported	Not reported	Not reported	Not reported	0.67-0.83

Carolan Doerflinger 2004	Adm	2.55	2.55	2.52	2.62	Not reported

Hommelstad and Ruland 2004	Nurses	2.2	2.8	2.5	2.6	0.67-0.74

Glacken and Chaney 2004	RN	2.54	3.09	2.31	2.64	Not reported

Patiraki *et al. *2004	Nurses	2.18	2.85	2.82	2.91	0.67-0.81

LaPierre *et al. *2004	Nurses	2.58	3.15	2.72	2.70	0.47-0.83

Nilsson Kajermo 2004	RN	2.2	2.8	2.1	2.6	0.69-0.83

Fink *et al. *2005	Pre Post	2.38 2.26	2.76 2.61	2.17 2.14	2.65 2.57	0.67-0.80 0.58-0.79

Ashley 2005	Critical care nurses	2.44	2.87	2.23	2.51	0.706-0.818

Baernholdt 2005	Chief govern-ment nursing officers	**1.42**	1.86	1.91	2.03	0.57-0.77

Karkos and Peters 2006	Nurses	2.25	2.63	2.12	2.48	Not reported

Thompson *et al. *2006	RN	Not reported	Not reported	Not reported	Not reported	0.63-0.84

Atkinson and Turkel 2008	RN	2.23	2.61	2.16	2.38	Not reported

Boström *et al. *2008	RN	2.19	2.71	2.17	2.62	0.67-0.78

Chau *et al. *2008	RN	2.63	3.00	2.63	2.74	0.71-0.93

Oh 2008	RN, NM	2.17	2.60	2.24	2.59	0.71-0.84

Brown *et al. *2009	Nurses	2.28	2.63	2.16	2.39	0.67-0.82

Schoonover 2009	RN	2.35	2.88	2.05	2.53	Not reported

Strickland and O'Leary-Kelly 2009	Educators	2.80	2.94	2.19	2.64	Not reported

RN = registered nurses, NM = nurse managers, Stud = Nurse students, Adm = administrators, CNS = clinical. specialist nurses

PreIG = pretest intervention group, PreCG = pretest control group, PostIG = posttest intervention group, PostCG = pretest intervention group.

The highest and lowest values on each subscale are bolded.

#### Content validity and response process

In 14 of the included studies, a pretest/pilot study was performed to test the items before the major study [23,30,36,38,44,51,52,55,56,62,63,66,69,78]. These pretest/pilot studies resulted in minor changes in wording of some items. In some of the pilot studies performed on translated versions of the scale, an item was added regarding use of the English language as a barrier.

#### Internal structure

In 13 studies, the authors performed factor analyses (Table 6). Of these, 10 resulted in three- to eight-factor solutions that differed more or less from the factors identified by Funk *et al. *[25,32,41,47,53,55,64,65,67,78]. The factor analyses performed by Hutchinson and Johnston [53], Ashley [41], and Mehrdad *et al. *[78] resulted in four factors that were almost identical to those identified by Funk *et al. *[16]. Dunn *et al. *[48] performed a confirmatory factor analysis and concluded that the factor model proposed by Funk *et al. *was not appropriate for their data.

**Table 6:** Factor analyses performed (n = 13).

Authors, year, country	Number of factors identified (no. of items included in the solution) Cronbach's alpha values of the factors	Variance accounted for by the factors %	Methods used
Funk et al. 1991, USA	4 (28) in both samples 0.65-0.80	43.4 respectively 44.9	Principal Component Analysis (PCA) with varimax rotation

Shaffer, 1994, USA	Several possible solutions were identified		Not reported

Dunn *et al. *1998, UK	The Funk model not appropriate		Confirmatory factor analyses (structural equation modeling)

Retsas and Nolan, 1999, Australia	3 (26)	38.9	PCA with varimax rotation

Retsas, 2000, Australia	4 (29) 0.68-0.85	46.5	PCA with varimax rotation

Marsh *et al. *2001, UK	4 (27 resp 24) The items loaded inconsistently on the four factors (two samples). Impossible to interpret the factors		PCA followed by confirmatory factor analysis

Closs and Bryar, 2001, UK	4 (23) 0.66-0.79	47.5	PCA with varimax rotation

Sommer, 2003, USA	8, 4, and 3 factors were possible solutions		Not reported

Hutchinson and Johnston, 2004, Australia	4 (27) 0.54-0.74	39.2	PCA

Kirshbaum *et al. *2004, UK	3		Least squares extraction with varimax rotation

Nilsson Kajermo, 2004, Sweden	4 (27) 0.90-0.96	45.3	PCA with varimax rotation

Ashley, 2005, USA	4 (29)	Not reported	PCA with varimax rotation

Mehrdad *et al. *2008, Iran	4 (31)	46.5	PCA

#### Associations between perceptions of barriers and other factors

In many studies, associations between demographic data--concerning, for example, age (n = 36), education (n = 38), and professional experience (n = 34)--and the perceptions of barriers were investigated. These findings were inconclusive. Furthermore, the demographic data were often presented in different ways and were correlated with the subscales or to the individual items of the BARRIERS scale, thus making it difficult to obtain a distinct picture of these associations.

### The main barriers to research utilization

In 84% (n = 53) of the 63 studies, the perceived barriers were presented in rank order, primarily based on the percentage of respondents agreeing with each item being a moderate or great barrier to research use. In many studies, all items were rank ordered, whereas in others, only the top ten, five, or three were presented. In five studies, the rank order was derived from the mean value of the items [57,63,72,77,83]. Some studies presented rank orders based on both the percentage of respondents agreeing with the item being a barrier and the mean values of each item [39,40,49,51,53,59,62,64,71,73,78,80,82]. In Table 1, the items of the BARRIERS scale are presented according to the original subscales. For each item, the range in percentage of respondents agreeing with the item being a great or moderate barrier is given as reported for each study. The items 'there is insufficient time on the job to implement new ideas,' 'the nurse does not have time to read research,' 'the nurse does not have enough authority to change patient care procedures,' 'the statistical analyses are not understandable,' together with 'the relevant literature is not compiled in one place' were most frequently reported among the top ten barriers (Table 1). Six of the ten top items belonged to the setting subscale. Four of the items in the BARRIERS scale were not among the top-ranked barriers in any of the studies (Table 1).

In 32 of the studies, the results were presented as mean values of the subscales (Table 5), with the highest values for the setting and presentation subscales. Higher values indicate greater perceived barriers. The main barriers to using research were related to the setting and how the findings are presented.

### Correlations between reported rank orders of the included studies

The rankings of barriers in the studies reporting all items (n = 37) were compared using Spearman's rank order correlation. This resulted in 703 correlation coefficients, ranging between -0.02 and 0.96. Of these, 461 correlation coefficients exceeded 0.50, and 485 correlations were found to be significant (p < 0.01). Thus, the rank orders of the included studies were correlated significantly (p < 0.01) with few exceptions, despite variations in wording of items, sample size, response rate, and study settings. The greatest exception was Baernholdt's study on government chief nursing officers internationally [23,26], in which the rank order correlated significantly (p < 0.01) with just one other study [63].

Researchers who studied the relationship between perceived barriers and use of specific research findings [23,44,50,51] reported, overall, the same top ten rank ordering of barriers as reported in other studies, with the exception of Baernholdt [23,26].

### Detecting changes in nurses' perceptions

In only two of the studies was the BARRIERS scale used at more than one time, in a pre- and post-intervention design [42,76]. Bobo [42] studied the impact of electronic distribution of nursing research, and Fink *et al. *[76] studied the impact of educational material and organizational strategies on nurses' perception of barriers to research utilization. Both studies found a significant decrease in the mean scores for two of the subscales (the 'nurse' and the 'setting' [76], and the 'nurse' and the 'presentation' [42], respectively) after interventions to support research utilization.

### Main barriers over time

To understand how the barriers have changed over time, the sample was arbitrarily divided into two groups; one group included studies published before 2000, and the other consisted of studies from 2000 onward. Subscale mean values for studies published before 2000 (n = 8) were: nurse 2.31, setting 2.62, research 2.15, and presentation 2.55, and the mean values for studies published during or after year 2000 (n = 23) were: nurse 2.35, setting 2.74, research 2.30, and presentation 2.57. We found no significant differences in mean values when comparing over time. We also explored the top ten items and found no significant differences over time in the percentage of nurses reporting the items as great or moderate barriers.

### Barriers in different geographic locations

We categorized the studies according to where they were performed, *i.e.*, North America (n = 26), European English-speaking countries (n = 12), European non-English-speaking countries (n = 12), and Australia and Asia (n = 7). We did not find any significant differences in mean subscale values when comparing across geographic locations.

With regard to the top ten barriers, we found significant differences (p < 0.01) for three of the top ten items when comparing mean percentages for agreement on an item being a barrier. Fewer nurses from European non-English-speaking countries reported 'the nurse is unaware of the research' as a barrier than did nurses from European English-speaking countries (34.2% versus 60.2% p = 0.005) or nurses from North America (34.2% versus 56.4%, p = 0.012). A higher percentage of nurses from European English-speaking countries and European non-English-speaking countries reported 'the facilities are inadequate for implementation' as a barrier than did nurses from North America (69.2%% versus 46.3%, p = 0.001, and 65.8% versus 46.3%, p = 0.006, respectively). For the item 'other staff are not supportive of implementation,' a higher percentage of nurses from European English-speaking countries perceived it as a barrier than did nurses from non-English-speaking countries in Europe (65.6% versus 43.7%, p = 0.006).

For 14 of the 15 studies performed in non-English-speaking countries, an extra item was included concerning the fact that most research is published in the English language, which is a foreign language to many respondents. This language item was among the top ten barriers in 11 of these studies [12,25,28,30,40,62,63,71,75,80,85].

### Associations between nurses' perceptions of barriers and reported research use

An important dimension of validity is the assessment of the hypothesized relationships between the scale items and a relevant outcome, in this case the anticipated association between barriers to research utilization and research use. However, few studies (n = 6) reported any attempt to examine an association between barriers and research use [12,24,43,60,66,73]. Of these, five reported only bivariate assessments and one used a multivariate assessment. Barta found no significant correlation between research use and reported barriers [24]. McCleary and Brown reported one significant subscale correlation, between research use and 'characteristics of the nurse,' suggesting that nurses reporting more research use perceived fewer barriers related to the nurse's research values, skills, and awareness [60]. Boström *et al. *reported a weak but significant correlation between the presentation subscale and research use [12]. In this study, the self-identified research users rated significantly lower on three subscales (presentation, nurse, and research) than did the non-research users. Brown *et al. *found two significant correlations between the presentation subscale and 1) knowledge and skills with evidence-based practice (EBP), and 2) practice of EBP, indicating that greater perceived barriers regarding the presentation of research were associated with lower perceived knowledge and skills and less use of EBP. The third association was between the setting subscale and knowledge and skills with EBP, revealing that the more the setting was perceived as a barrier, the lower the nurses' perceptions of their own knowledge and skills [73]. Brenner found no relationship between frequency of reading research journals and nurses' perceptions of barriers [43]. Shaffer, using path analysis, found that research activities, such as the reading of research journals, did not affect nurses' perceptions of barriers [66].

## Discussion

Assessing over 60 studies using the BARRIERS scale, we found reported barriers to research use have remained constant over time and across geographic locations. The rank order of items was found to be uniform, although the percentage of agreement varied between studies. Despite differences in method, our findings were similar to those of Carlson and Plonczynski [22], who analyzed correlations between year of publication and mean percentage of items reported as barriers to research use. They concluded that perceived barriers have not changed since the scale's publication. Conversely, we compared the mean values of the four subscales between two groups (1991 to 1999 and 2000 to 2009) using Student's t-test and did not find any significant differences when compared across time. Using this approach, we confirmed Carlson and Plonczynski's [22] findings. There are some minor differences between our results and Carlson and Plonczynski's [22] when comparing across geographic locations. Carlson and Plonczynski [22] compared barriers across three geographic locations: United States of America, United Kingdom, and other countries. Using vote counting to calculate differences between countries, they found differences on five items. We compared barriers across geographical locations by dividing the studies based on whether they included subjects from North America, Europe-English, Europe non-English, or Australia/Asia. Using ANOVA and Bonferroni *post hoc *tests to compare mean percentages for the top ten items and the subscale means, we did not find any differences in subscale means, but did find three differences across the top ten items. Both our results and Carlson and Plonczynski's suggest that a significantly higher percentage of nurses outside North America view inadequate facilities as a barrier to research use than do their North American colleagues.

The quality of the 63 studies was generally weak to moderate (22 weak, 38 moderate, and 3 strong), reflecting trends often reported in systematic reviews. We found no differences in reported findings between the weak and stronger studies, however, possibly suggesting that the general and descriptive nature of the studies was resistant to methodological flaws. Nonspecific wording limits the usefulness of the BARRIERS scale as a tool for planning interventions. For example, the statement 'facilities are not adequate for implementation,' one of the top ten items, provides little insight into aspects of facilities that might be deficient. Facilities could refer to material resources, such as access to a computer and electronic databases, or to human resources, such as access to clinical specialists or facilitators. Nonspecific barrier items could contribute to the consistent results. Additionally, two consistently high-ranking items ('lack of time to read' and 'lack of time to implement research') require further investigation if they are to be used to plan interventions. The meaning of 'time' as a barrier to research use is rarely described and is not described in the scale. Time is a complex phenomenon and, as Thompson *et al. *recently suggested, busyness, in the context of research utilization, includes multiple dimensions such as physical time, but perhaps more importantly, mental time [86]. Such a distinction has important implications for designing strategies to overcome barriers to research use. Additionally, study authors using the BARRIERS scale relied almost exclusively on cross-sectional designs. This approach is problematic when exploring complex barriers such as time. Tydén suggested that using a longitudinal design to study research utilization provides more accurate findings [87]. Using a longitudinal design to study environmental and health officers, he found that respondents initially reported socially acceptable barriers (such as lack of time), but as the study proceeded, respondents changed their responses to reflect more complex underlying barriers [87]. Another approach was used by Ashley, who asked nurses to rank barriers in relation to a specific research utilization project and found that time was not ranked among the top three barriers [41].

Despite minor modifications of the BARRIERS scale across studies, our results support the reliability of the BARRIERS scale; that is, the reported Cronbach's alpha values indicate internal consistency. However, the validity of the scale to accurately capture barriers to research use is much more at issue. This instrument, developed in accordance with healthcare environments in the late 1980s and early 1990s, has been administered predominantly in its original format since then, without detecting any changes in the perceptions of barriers over time. Both healthcare systems and the nursing profession have undergone significant changes over the past 30 years, and it is difficult to believe that such changes have not affected nurses' reported perceptions of barriers to research use. For example, in healthcare today, patient participation in decision making is much more evident and, in some countries, even legally regulated. Patients' preferences and opinions could, hypothetically, present a barrier to research use. Barriers with respect to patients' opinions were added to the BARRIERS scale by Greene, who measured barriers toward pain management in oncology care [51]. 'Patients will not take medication or follow the recommendations' was rated as the third highest ranked barriers by the nurses.

In addition to changes in patient participation in healthcare decision-making, dramatic advances have occurred in information technology and its use in healthcare. Hutchinson and Johnston [21] identified information technology as a mechanism for supporting point-of-care retrieval of research. Additionally, organizations such as the Cochrane Collaboration provide online access to synthesized research evidence. It stands to reason that efforts to increase accessibility to synthesized research evidence would lead to a decrease in the percentage of nurses reporting barriers related to presentation of research. However, despite these recent advances aimed at making research more accessible to practitioners, the item 'the relevant literature is not compiled in one place' and the presentation subscale remain among the top items and subscales, respectively.

Items within the research subscale, and the research subscale itself, were not among the top barriers in any of the studies (Table 1). The research subscale items in the BARRIERS scale do not reflect innovation characteristics as reported in Rogers' diffusion of innovation theory. Rogers identified relative advantage, compatibility, complexity, observability, and trialability of the innovation, as well as the user's values and experiences of the innovation [18], as key attributes to adoption of innovation. However, the items in the research subscale refer primarily to the quality of the research (Table 1). There is evidence to suggest the quality of research plays a minimal role in influencing nurses to use or not use research. Instead, factors related to compatibility and trialability are of greater importance [88]. One would therefore expect that this subscale would be of limited usefulness and that efforts would be better spent investigating attributes more closely aligned with Rogers' attributes of successful innovations.

An untested assumption of the BARRIERS scale is that a relationship exists between perceptions of barriers to research utilization and actual research use. Of the 63 studies in the present review, only six studies [12,24,43,60,66,73] investigated this relationship. Of these, three studies found significant bivariate correlations between research use and perceived barriers to research use. Specifically, research use was associated with fewer barriers in relation to nurses' research values, skills, and awareness [60], and with respect to the presentation of research [12,73]. Further, Brown *et al. *found a significant negative association between perceptions of barriers in the setting and nurses' knowledge and skills in using research [73]. While this finding may point to a potential link between barriers in the setting and research use, there is no evidence of such a relationship. Potential associations cannot be asserted on the basis of correlations that, when subjected to more rigorous multivariate assessments, often lose statistical significance. Thus, despite our finding that the setting represents the greatest perceived barrier to research use, a significant relationship between this subscale and actual research use has not been reported, leaving significant unanswered questions regarding the scale's validity.

Continued reliance on the BARRIERS scale to elicit perceptions of barriers to research uptake is unlikely to provide an accurate picture of the barriers that exist in the current clinical setting. Recent work undertaken by the Cochrane Effective Practice and Organisation of Care Group (EPOC) provides alternative approaches to categorizing and assessing potential barriers to research use [13]. The EPOC Group classified barriers into eight categories: information management and clinical uncertainty, sense of competence, perceptions of liability, patient expectations, standards of practice, financial disincentives, administrative constraints, and others [13]. A similar approach is taken by Gravel *et al.*, who present a comprehensive taxonomy of barriers and facilitators to shared decision making that could readily be applied to research use [89].

### Strengths and limitations

There are limitations to this systematic review. First, we did not exclude studies based on quality, as we were interested in comparing results from as many studies as possible to capture possible differences. Second, heterogeneity between the studies in terms of reporting results led to complicated data extraction procedures, preventing meta-analysis. Third, judgments related to data extraction and quality assessment create a certain amount of subjectivity that may influence the results. Finally, we included studies in English and Scandinavian languages only, and it is possible we missed potentially relevant studies published in other languages. Conversely, the review has several strengths. Since the previous review [22], 18 new articles were identified, strengthening the findings and conclusions of this present review. We used statistical analyses to compare barriers across time and geographical locations as well as to compare rank orders of perceived barriers of the included studies.

### Recommendation for future research

The key issue raised by this review is whether barriers to research utilization should be measured on a general and nonspecific level, or if specific barriers capturing both the context and the particular characteristics of the evidence (or innovation) should be assessed. We recommend that no further descriptive studies using the BARRIERS scale be undertaken, because further use would constitute a waste of scarce research resources. Instead, we recommend examination of various contextual and human factors for enhancing research use in a given organizational context. To advance the field and improve the quality of care for patients, tailored interventions need careful evaluation. Such interventions must address locally relevant barriers to research utilization and the characteristics of the intervention.

## Summary

The aim of this systematic review was to examine the state of knowledge resulting from use of the BARRIERS scale and, secondarily, to make recommendations about future use of the scale. Despite variations in study setting, sample size, response rate, assessed quality, wording of items, and the placement of the 'no opinion' response option, the rank orders of barriers were remarkably consistent in the studies we reviewed. The BARRIERS scale is a general (nonspecific) tool for identifying barriers to research use, and while reliable, little evidence supporting its construct validity exists. It has not been used to identify barriers to inform the development of strategies and interventions to promote research use. Thus, there is no evidence that the scale is useful for informing intervention studies. Furthermore, given the highly general nature of the items on this scale, it is unlikely that it has the ability to adequately inform interventions intended to increase the use of evidence in practice.

## Competing interests

The authors declare that they have no competing interests.

## Authors' contributions

All authors contributed to the design of the study and approved the submitted draft. DT performed the database searches. KNK and AMB reviewed and abstracted the articles and analyzed the data. All authors read and approved the final manuscript.
